# Optimal Deep Stacked Sparse Autoencoder Based Osteosarcoma Detection and Classification Model

**DOI:** 10.3390/healthcare10061040

**Published:** 2022-06-02

**Authors:** Bahjat Fakieh, Abdullah S. AL-Malaise AL-Ghamdi, Mahmoud Ragab

**Affiliations:** 1 Information Systems Department, Faculty of Computing and Information Technology, King Abdulaziz University, Jeddah 21589, Saudi Arabia; bfakieh@kau.edu.sa (B.F.); aalmalaise@kau.edu.sa (A.S.A.-M.A.-G.); 2 Information Systems Department, HECI School, Dar Alhekma University, Jeddah 22246, Saudi Arabia; 3 Center of Excellence in Smart Environment Research, King Abdulaziz University, Jeddah 21589, Saudi Arabia; 4 Information Technology Department, Faculty of Computing and Information Technology, King Abdulaziz University, Jeddah 21589, Saudi Arabia; 5 Department of Mathematics, Faculty of Science, Al-Azhar University, Naser City, Cairo 11884, Egypt; 6 Centre for Artificial Intelligence in Precision Medicines, King Abdulaziz University, Jeddah 21589, Saudi Arabia

**Keywords:** osteosarcoma, computer aided diagnosis, medical images, deep transfer learning, image processing

## Abstract

Osteosarcoma is a kind of bone cancer which generally starts to develop in the lengthy bones in the legs and arms. Because of an increase in occurrence of cancer and patient-specific treatment options, the detection and classification of cancer becomes a difficult process. The manual recognition of osteosarcoma necessitates expert knowledge and is time consuming. An earlier identification of osteosarcoma can reduce the death rate. With the development of new technologies, automated detection models can be exploited for medical image classification, thereby decreasing the expert’s reliance and resulting in timely identification. In recent times, an amount of Computer-Aided Detection (CAD) systems are available in the literature for the segmentation and detection of osteosarcoma using medicinal images. In this view, this research work develops a wind driven optimization with deep transfer learning enabled osteosarcoma detection and classification (WDODTL-ODC) method. The presented WDODTL-ODC model intends to determine the presence of osteosarcoma in the biomedical images. To accomplish this, the osteosarcoma model involves Gaussian filtering (GF) based on pre-processing and contrast enhancement techniques. In addition, deep transfer learning using a SqueezNet model is utilized as a featured extractor. At last, the Wind Driven Optimization (WDO) algorithm with a deep-stacked sparse auto-encoder (DSSAE) is employed for the classification process. The simulation outcome demonstrated that the WDODTL-ODC technique outperformed the existing models in the detection of osteosarcoma on biomedical images.

## 1. Introduction

Osteosarcoma is considered to be an aggressive bone malignancy which occurs often in the extremes of adolescents and children with a terminal diagnosis [1]. The occurrence of osteosarcoma is assumed to be the most frequent amongst all of the basic malicious tumors across the globe, achieving three cases per million people per year, including a female male ratio of 1:1.5. Meanwhile, the chance of living with this tumor is lesser. The five-year survival rate of patients affected by osteosarcoma was below 20% before the 1980s [2]. This type of tumor consists of a higher extent of malignity and is vulnerable to the lungs and metastases; as such, its medication seems to be very complex. Initial identification and medication could improve the survival rate of patients and decrease the chances of amputation [3]. In clinical prognoses, MRI images cannot pose significant biological and radiation hazards to the tissue in the course of inspection and are very apparent in tissue elements, such as the blood vessels and tumors [4]. This is why we usually utilize MRI images for detecting osteosarcomas. In the recent identification procedure of osteosarcoma MRI images, patients having osteosarcoma would produce a huge volume of image data, since the ratio of useful images is less. Each osteosarcoma patient will produce 600 to 700 images, but the fact is just 10 to 20 images are useful for medication, as incomplete statistics are reported [5]. Nowadays, preliminary processing and screening of raw pictures are conducted by physicians manually [6]. This procedure includes more material sources and labor forces. Meanwhile, there is no standard interpretation the diagnostic features of osteosarcoma.

The computer-aided diagnosis (CAD) of osteosarcoma metastasizes a prominent research focus in the literature, as it is helpful to the physician in detecting the nodule in a lung of patients at the prior level [7]. There are several methodologies that have been suggested in recent decades. An effective ML tool is the Convolutional Neural Network (CNN), as it can be trained on labeled data or images which are passed into the network for attaining output. It is mainly used for image classification. It derives the features and learns essential features in the convolutional layers, then categorizes the output as the presence or absence of osteosarcoma metastasizes by the fully connected layers [8]. This learning technique, known as a supervised learning algorithm, can be employed for training the CNN model to accomplish effective results by the use of high-quality images in the dataset [9,10]. 

This study develops a wind driven optimization with deep transfer learning enabled osteosarcoma detection and classification (WDODTL-ODC) method. The presented WDODTL-ODC model intends to determine the presence of osteosarcoma in the biomedical images. To accomplish this, the osteosarcoma model involves Gaussian filtering (GF) based on pre-processing and contrast enhancement techniques, and is followed by deep transfer learning using SqueezNet model utilized as a feature extractor. At last, the WDO algorithm with deep-stacked sparse auto-encoder (DSSAE) is employed for the classification process. The simulation outcome of the WDODTL-ODC technique is tested using benchmark biomedical images.

## 2. Related Works

Shen et al. [11] suggest that an osteosarcoma-supported segmentation methodology depends on a directed combined bilateral network (OSGABN) that improvises the segmentation precision of the model and hugely diminishes the variable scale, efficiently easing the issues which are discussed above. The rapid bilateral segmentation network (FaBiNet) is utilized for the division of images. It is a high accuracy method that has a detailed branch, which takes a lightweight semantic branch and low-level data, and which takes high level semantic contexts. Varalakshmi et al. [12] recommend an original route for classifying distinct osteosarcoma forms having a higher precision by utilizing histology image (Microscopic bone image). The examination of bone utilizing histology is a prolonged and tiresome procedure. During this article, the eXtreme Gradient Boosting (XGBoost) system is utilized for classifying osteosarcoma.

The researchers in [13] suggest an original CNN structure made up of Concatenation of numerous Networks, termed as C-Net, for categorizing biomedical images. The method inculcates numerous CNNs involving Inner, Outer, and Middle. The initial 2 portion of the structure consists of 6 networks which act as feature extractors for feeding into the Inner network to categorize the images with regard to benignancy and malignancy. Asito et al. [14] suggest a computer aided prognosis system depends on CNNs for the recognition of osteosarcoma on bone radiographs. The CNN must denote areas of the image which can consist of tumors. For indicating such areas on the image, we suggest dividing image into windows and categorizing them individually with the help of CNN. Methods for pre-processing, like labeling and window exclusion, are recommended. In the suggested system comparison is made for 2 CNNs. 

Asmaria et al. [15] mainly focus to categorize the cell viability of osteosarcoma’s dataset with hematoxylin and eosin (H&E) stained. The CNN structure incorporated 6 convolution layers, fully linked layers, and max pooling layers to feature extraction. Data augmentation is utilized for boosting the executions. Sharma et al. [16] discover the better appropriate edge detection system once the 2 feature sets one without hog and another with hog is ready. For testing the effectiveness of such feature sets, the Random Forest, 2 ML methods, and support vector machine (SVM) and, are used. 

Rajagoal et al. [17] reveals several methods of medical image processing and DL and implies them to identify and categorize tumors as malignant or benign. Approaches utilized inculcates image pre-processing utilizing filtering methodologies, K-means edge detection, and segmentation for identifying cancerous areas in Computer Tomography (CT) images for enchondroma, osteochondroma, and Parosteal osteosarcoma, forms of bone cancer. Once the process of segmentation of the tumor is done, categorization of cancerous cells and benign is made by using a DL method related CNN classifier. Few works have been available in the literature for osteosarcoma classification and design of automated classification models need to be explored more. In addition, the existing models do not focus on the hyper-parameter selection process, which mainly influences the performance of the classification model. Particularly, the hyper-parameters such as epoch count, batch size, and learning rate selection are essential to attain effectual outcome. Since the trial and error method for hyper-parameter tuning is a tedious and erroneous process, metaheuristic algorithms can be applied. Therefore, in this work, we employ WDO algorithm for the parameter selection of the DSSAE model. 

## 3. The Proposed Model

**Figure 1:**
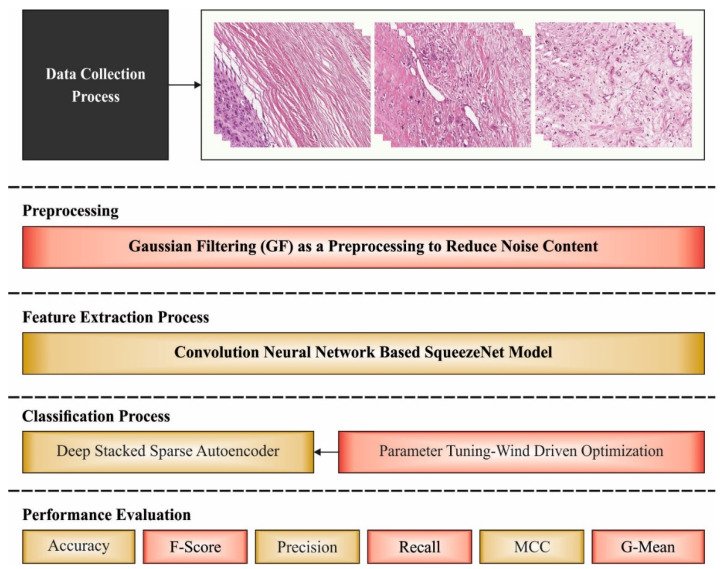
Overall process of WDODTL-ODC technique.

In this study, a novel WDODTL-ODC approach was established to determine the presence of osteosarcoma in biomedical images. The WDODTL-ODC technique comprises GF-based pre-processing and contrast enhancement techniques. In addition, deep transfer learning using SqueezNet model is utilized as feature extractor. At last, the WDO algorithm with DSSAE is employed for classification process. Figure 1 depicts the overall process of WDODTL-ODC technique.

### 3.1. Image Pre-Processing

At the primary level, the WDODTL-ODC technique comprises GF-based pre-processing and contrast enhancement techniques. GF is a method that decreases pixel difference by weighted average for image smoothing from different applications. But, the low--pass filter could not preserve image detail, for example, the textures and edges. Then, linear translation—variant function f defines the abovementioned filtering procedure as follows [18]:

(1) $$f\left(p\right)=\underset{q}{\sum }K_{p,q}\left(Q\right)P_{q}$$

In Equation (1), $K_{p,q}$ signifies the pixel q centred at pixel p in filter kernel K, and Q and P denote guidance and input images, correspondingly. For instance, the kernel of Bilateral Filter (BF) is defined as:

(2) $$K_{p,q}\left(Q\right)=\frac{1}{n}\;\exp\;\left(-\frac{\|p-q{\|}^{2}}{\sigma_{s}^{2}}\right)\cdot \exp\;\left(-\frac{\|P_{p}-Q_{q}{\|}^{2}}{\sigma_{r}^{2}}\right)$$

The exponential distribution function is utilized in Equation (2) to compute the effect of spatial distance by $\exp\;\left(-\frac{\|p-q{\|}^{2}}{\sigma_{r}^{2}}\right)$, and $\exp\;\left(-\frac{\|P_{p}-Q_{q}{\|}^{2}}{\sigma_{r}^{2}\;}\right)$ describes the influence of pixel intensity range. If *Q* and *P* are identical, Equation (2) is simplified as a single image smoothing form.

### 3.2. Feature Extractor

**Figure 2:**
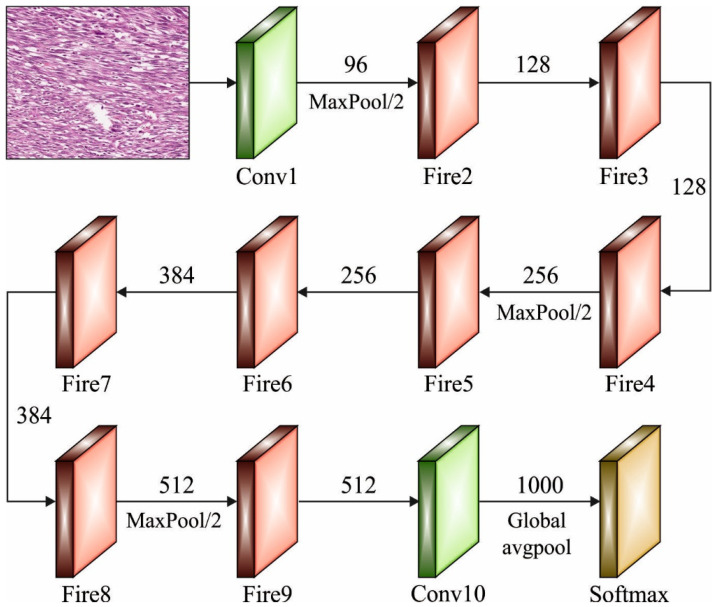
Structure of SqueezeNet Model.

Next to image pre-processing, deep transfer learning using SqueezNet model is utilized as feature extractor. SqueezeNet is an efficient and lightweight CNN model [19]. With SqueezeNet, we achieve a 50× reduction in model size compared to AlexNet, while meeting or exceeding the top-1 and top-5 accuracy of AlexNet. The SqueezetNet model achieves effective performance with less number of parameters. There are two important parts of CNN, namely feature extraction and classification. The extracted features were utilized for the accurate classification of images. Specifically, these 2 parts of CNN achieve the initial function of CNNs. The feature extracting part of CNN contains convolutional and sampling layers. The filter of convolutional layer was utilized for diminishing the noise in the images; afterward, the feature of images was improved. The convolutional procedure was complete amongst the upper layer feature vector and the presentation layer convolutional kernel layer. The activation function of CNN lastly finalizes the convolutional procedure computations. The efficacy of training a NN was obvious when utilizing the cost function that represents the proportion amongst the trained instance and the reached output. Figure 2 illustrates the structure of SqueezeNet technique.

(3) $$Z=\frac{-1}{m}\sum^{\;}\left[x\;ln\;a\left(1-x\right)\ln\left(1-\beta \right)\right]$$

whereas m represents the amount of trained data, x signifies the predictable value, and β denotes the actual value in the resultant layer. 

The activation function roles a vital part in the classifier procedure with transmission kernel size and weighted the resultant of CNN method. The *ReLU* activation function was between the generally utilized activation function. It can be approximately utilized from every CNN technique for setting every negative value equivalent to 0. These zero settings inhibit many nodes in participate from the learning procedure. Another function, namely *LReLU* and *ELU* that offer smaller negative values was rarely utilized from classifier approaches. *ReLU* activation function displays optimum outcomes than *LReLU* activation function from the classifier which is utilized in our classifier method. The provided formula mathematically represents the *ReLU* activation function.

(4) ReLU(x)=max(0; x)

It is mostly employed in an embedded setting; it includes different methodologies of compression model. For instance, 3×3 convolutional kernel in the presented model is substituted with 1×1 convolutional kernel. With this method, the parameter count for single convolutional function is minimized by the factor of 9. Additionally, the 3×3 convolutional kernel is decreased as well as down-sampling is delayed in the network layer. Consequently, the presented model decreases the number of trained variables and the computation efforts. Therefore, it is feasible to set up SqueezeNet in memory-limited hardware device. In comparison to current AI models, we have observed that SqueezeNet has the minimum parameter count; therefore, it is the right selection for robot vacuum applications. Unfortunately, the model size (viz., 6.1 MB) is greater when compared to the memory space presented in robot vacuums. 

### 3.3. Image Classification

At the final stage, the WDO algorithm with DSSAE is employed for classification process. The DDSAE model is applied in this work as it learns non-linear transformations with a non-linear activation function and multiple layers. Besides, it is effective in eLearning different layers with an auto-encoder (AE) instead of learning one huge transformation with PCA. The essential component of DSSAE is the AE [20], viz., is a standard NN that learns to map the input Y to output Z. The AE is classified into an encoding part $\left(W_{Y},B_{Y}\right)$ that map input Y to code C, and a decoding part $\left(W_{Z},B_{Z}\right)$ that map code C to recreated dataset Z,

(5) $$\begin{array}{c} Y \end{array}\;\begin{array}{c} encoder\\ \mapsto \end{array}\begin{array}{c} C \end{array}\;\begin{array}{c} decoder\\ \mapsto \end{array}\begin{array}{c} Z \end{array}\;$$

Given that output, Z is equivalent to the input Y.

Whereby the encoded part is weighted $W_{Y}$ and bias $B_{Y}$, and the decoded part is with weight $W_{Z}$ and bias $B_{Z}$.

(6) $$C=g_{LS}\left(W_{Y}Y+B_{Y}\right)$$

(7) $$Z=g_{LS}\left(W_{Z}C+B_{Z}\right)$$

From the equation, the output Z is an approximation of input Y, and $g_{LS}$ indicates the log sigmoid function:

(8) $$g_{LS}\left(x\right)=\frac{1}{1+\exp\left(-x\right)}$$

The SAE is variant of AE. In order to minimalize the errors among the input vector Y as well as output Z, the loss function of AE is assumed as follows:

(9) $$I_{AE}\left(W_{Y},\;W_{Z},B_{Y},B_{Z}\right)=\frac{1}{N_{S}}\|Z-Y{\|}^{2}$$

In Equation (9), $N_{s}$ indicates the number of training instances. From Equations (6) and (7), the output Z is formulated by using following equation

(10) $$Z=g_{AE}\left(Y|W_{Y},\;W_{Z},B_{Y},B_{Z}\right)$$

In Equation (10), $g_{AE}$ indicates the abstract of AE; Thus, Equation (9) is written by

(11) $$I_{AE}\left(W_{Y},\;W_{Z},B_{Y},B_{Z}\right)=\frac{1}{N_{S}}\|g_{AE}(Y|W_{Y},\;W_{Z},B_{Y},B_{Z})-Y{\|}^{2}\;$$

Actually, $L_{2}$ regularization term $\Gamma_{w}$ of the weight $\left(W_{Y},\;W_{Z}\right)$ and $\Gamma_{s}$ regularization terms of the sparsity constraints are determined to evade trivial or overcomplete mapping.

(12) $$l_{SAE}\left(W_{Y},W_{Z},B_{Y},B_{Z}\right)=\frac{1}{N_{S}}\|g_{AE}(Y|W_{Y},W_{Z},B_{Y},B_{Z})-Y{\|}^{2}+c_{s}\times \Gamma_{s}+c_{w}\times \Gamma_{w}$$

In Equation (12), $c_{s}$ indicates the sparsity regulation factor, $c_{w}$ represent the weighted regulation factors. The sparsity regularization term is determined by:

(13) $$\Gamma_{s}=\sum_{j=1}^{\left|C\right|}g_{KL}\left(\rho ,{\hat{\rho }}_{j}\right)=\sum_{j=1}^{\left|C\right|}\rho \;\log\;\frac{\rho }{{\hat{\rho }}_{j}}+\left(1-\rho \right)\log\;\frac{1-\rho }{1-{\hat{\rho }}_{j}}$$

In Equation (14), $g_{KL}$ represent the Kullback-Leibler divergence function, |C| denotes the element count of internal code output C. $\hat{\rho }$ indicates the j−th average activation value on each $N_{S}$ trained sample and ρ denotes chosen value, called sparsity proportion factor. The $\Gamma_{w}$ weight regularization term is determined by

(14) $$\Gamma_{w}=\frac{1}{2}\times \|W_{Y}W_{Z}{\|}_{2}^{2}.\;$$

The training process is fixed to scaled conjugate gradient descent (SCGD) technique. It can be utilized SAE as structure block and generate the last DSSAE classification with subsequent 3 functions: (i) It can contain PZM layer, input layer, vectorization layer, and pre-processing layer; (ii) It can be stack 4 SAEs with several amounts of hidden neurons; (iii) It can be attached softmax layer at the end of AI method.

### 3.4. Parameter Optimization

To tune the parameters related to the DSSAE model, the WDO algorithm has been exploited. Zikri Bayraktar, in 2010, first developed the concept of WDO [21]. It is inspired by the natural wind movement concept that serves as a stabilizer for equalizing the air pressure inequality. Wind blow from a higher-to lower-pressure region, along with a velocity viz. directly proportionate to pressure gradient (the high the pressure variance, the strong the wind blows) [22]. The major concept behind the proposed algorithm is Newton’s 2nd law of motion:

(15) $$\rho \vec{a}=\sum^{\;}\vec{F_{i}}$$

In Equation (15), the acceleration vector can be expressed by the term a, the air density for a component with smaller amount is represented as ρ, and the force acting on the mass is denoted by $F_{j}$. Temperature, Air pressure, and density are interrelated with the formula based on the ideal gas law formulated in the following

(16) P=ρRT

In Equation (16), R, and T are correspondingly represented as pressure, universal gas constant, and temperature.

In Equation (15), the important force that causes the wind blows in a direction to diverge in that direction is classified into frictional force $\left(F_{F}\right)$, pressure gradient force $\left(F_{PG}\right)$, coriolis force $\left(F_{C}\right)$ and gravitational force (F).

(17) $${\vec{F}}_{PG}=-\nabla P\delta V$$

(18) $${\vec{F}}_{C}=-2\Omega \times \vec{u}$$

(19) $${\vec{F}}_{G}=\rho \delta \vec{g}$$

(20) $${\vec{F}}_{F}=-\rho \alpha \vec{u,}$$

From the above equations, the pressure gradient is represented as ∇P, δV indicates a small volume of air, Ω signifies the rotation of earth, u denotes the wind velocity vector and g represents the gravitational acceleration. The fusion of Equations (15) and (17)–(20) generate an expression given in the following:

(21) $$\rho \vec{u}\Delta t=-\nabla P\delta V-2\Omega \times \vec{u}+\rho \delta V\vec{g}-\rho \alpha \vec{u}.\;$$

The unit step of time, Δt is equivalent to 1. The fusion of Equations (16) and (21) generated an equation represented in the Equation (22):

(22) $$\begin{array}{r} {\vec{u}}_{new}=-g{\vec{x}}_{old}+\left(1-\alpha \right){\vec{u}}_{old}+\left|1-\frac{P_{\;\max\;}}{P_{old}}\right|\\ RT\left(x_{\;\max\;}-x_{old}\right)-\frac{cu_{old}^{other\;dim}}{P_{old}} \end{array}$$

Let $u_{new}$ and $u_{old}$ be the upgraded velocity and the present velocity, correspondingly; $x_{old}$ and $x_{\max\;}$ indicates the existing position and the maximum pressure position of the air pack, correspondingly; $P_{\max\;}$ and $P_{old}$ indicates the maximal pressure and the pressure at the existing position, correspondingly; T indicates the temperature; and R, α, and c denote constant.

In Equation (22) the pressure value is generally higher. Thus, the velocity estimation is also higher. This causes the efficiency level of the WDO to reduce. Equation (22) is reordered into the subsequent formula:

(23) $${\vec{u}}_{new}=-g{\vec{x}}_{old}+\left(1-\alpha \right){\vec{u}}_{old}+\left|1-\frac{1}{k}\right|RT\left(x_{\;\max\;}-x_{old}\right)-\frac{cu_{old}^{other\;dim}}{k}.\;$$

In Equation (23), k signifies the ranking among each air parcel (k=1,2,…, 20). The subsequent formula is utilized for updating the location of air pack:

(24) $${\vec{x}}_{new}={\vec{x}}_{old}+{\vec{u}}_{new}\times \Delta t.\;$$

The WDO grows a fitness function (FF) for achieving higher classifier efficiency. It defines a positive integer for denoting the best efficiency of candidate solution. During this study, the minimized classification error rate was regarded as FF as existing in Equation (25).

(25) $$fitness\left(x_{i}\right)=ClassifierErrorRate\left(x_{i}\right)=\frac{number\;of\;misclassified\;samples}{Total\;number\;of\;samples}\times 100\;$$

## 4. Results and Discussion

**Figure 3:**
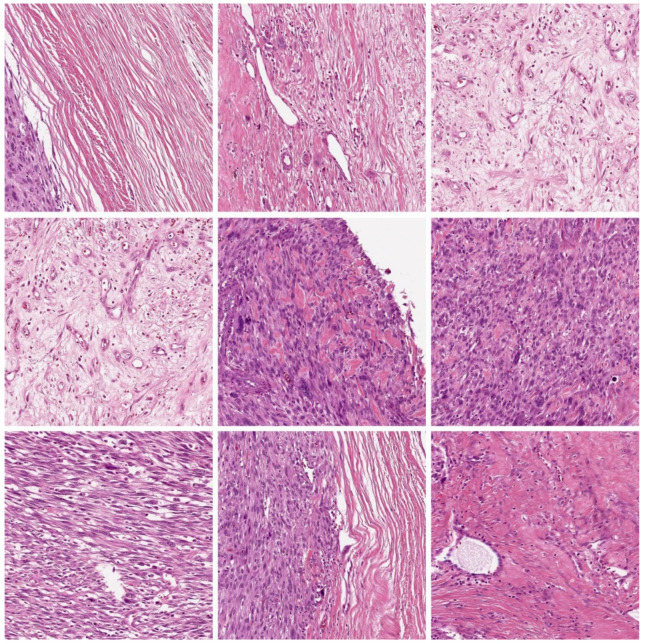
Sample Images.

The performance validation of the WDODTL-ODC model is tested using a benchmark dataset [23] comprising 1144 images under three classes. It includes 345 images under viable tumor (VT), 263 images under non-Viable Tumor (NVT), and 536 images under Non-Tumor (NT) class. A few sample images are depicted in Figure 3.

**Figure 4:**
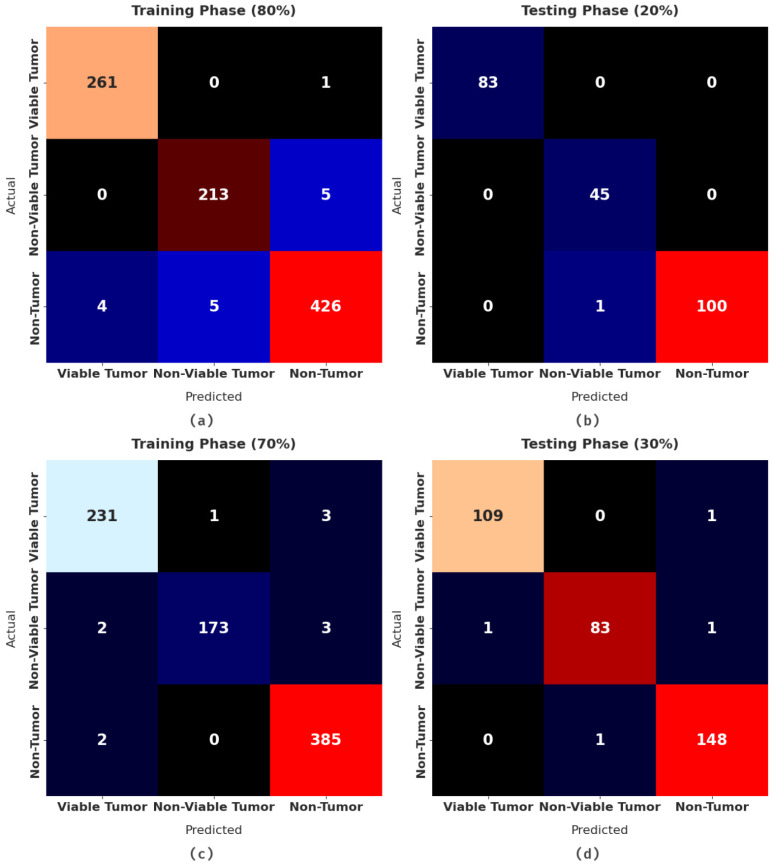
Confusion matrices of WDODTL-ODC technique (**a**) 80% of TR data, (**b**) 20% of TS data, (**c**) 70% of TR data, and (**d**) 30% TS data.

Figure 4 demonstrates the confusion matrices produced by the WDODTL-ODC model on distinct sizes of data. With 80% of TR data, the WDODTL-ODC model has categorized 261, 213, and 426 samples under VT, NVT, and NT classes respectively. Meanwhile, with 20% of TS data, the WDODTL-ODC approach has categorized 83, 45, and 100 samples under VT, NVT, and NT classes correspondingly. Eventually, with 70% of TR data, the WDODTL-ODC system has categorized 231, 173, and 385 samples under VT, NVT, and NT classes correspondingly. 

**Figure 5:**
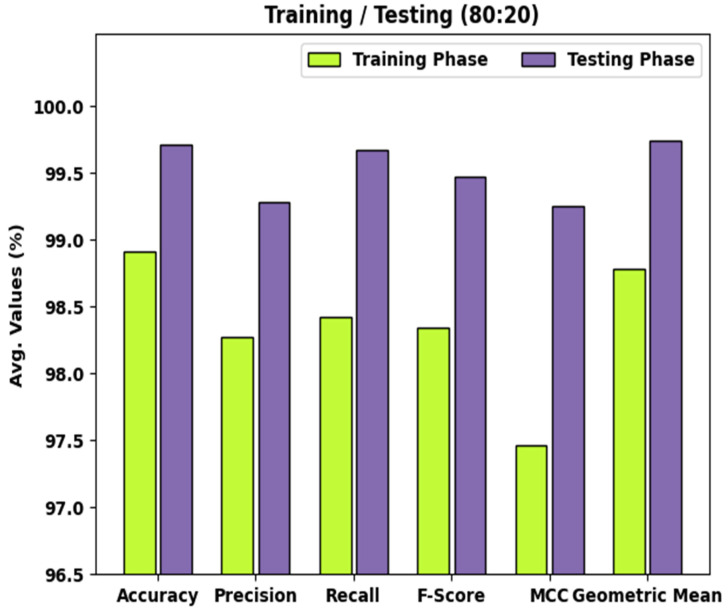
Result analysis of WDODTL-ODC technique under 80% of TR and 20% of TS data.

Table 1 and Figure 5 represent the detailed classifier results of the WDODTL-ODC model with TR data of 80% and TS data of 20%. The results implied that the WDODTL-ODC model has effectually categorized the images with maximum outcome. For instance, the WDODTL-ODC model has identified samples under VT class with $accu_{y}$, $prec_{n}$, $reca_{l}$, $F_{score}$, MCC, and $G_{mean}$ of 99.45%, 98.49%, 99.62%, 99.05%, 98.67%, and 99.50% respectively. Along with that, the WDODTL-ODC approach has identified samples under NVT class with $accu_{y}$, $prec_{n}$, $reca_{l}$, $F_{score}$, MCC, and $G_{mean}$ of 98.91%, 97.71%, 97.71%, 97.70%, 96.99%, and 98.49% correspondingly. Moreover, the WDODTL-ODC algorithm has identified samples under NT class with $accu_{y}$, $prec_{n}$, $reca_{l}$, $F_{score}$, MCC, and $G_{mean}$ of 98.36%, 98.61%, 97.93%, 98.27%, 96.71%, and 98.34% correspondingly. 

**Figure 6:**
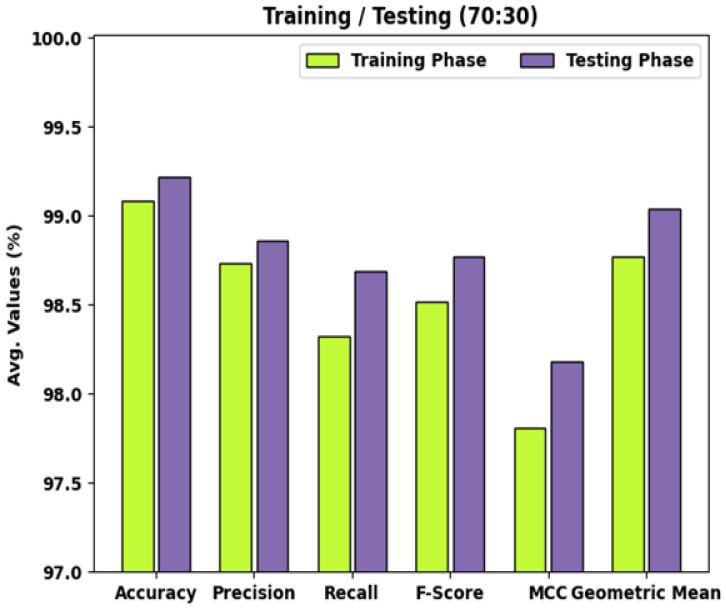
Result analysis of WDODTL-ODC technique under 70% of TR and 30% of TS data.

Table 2 and Figure 6 demonstrate the detailed classifier outcomes of the WDODTL-ODC algorithm with TR data of 80% and TS data of 20%. The results outperformed that the WDODTL-ODC algorithm has effectually categorized the images with maximal outcomes.

For instance, the WDODTL-ODC system has identified samples under VT class with $accu_{y}$, $prec_{n}$, $reca_{l}$, $F_{score}$, MCC, and $G_{mean}$ of 99%, 98.30%, 98.30%, 98.30%, 97.59%, and 98.79% correspondingly. Besides, the WDODTL-ODC system has identified samples under NVT class with $accu_{y}$, $prec_{n}$, $reca_{l}$, $F_{score}$, MCC, and $G_{mean}$ of 99.25%, 99.43%, 97.19%, 98.30%, 97.83%, and 98.51% correspondingly. Furthermore, the WDODTL-ODC approach has identified samples under NT class with $accu_{y}$, $prec_{n}$, $reca_{l}$, $F_{score}$, MCC, and $G_{mean}$ of 99%, 98.47%, 99.48%, 98.97%, 98%, and 99.01% correspondingly.

**Figure 7:**
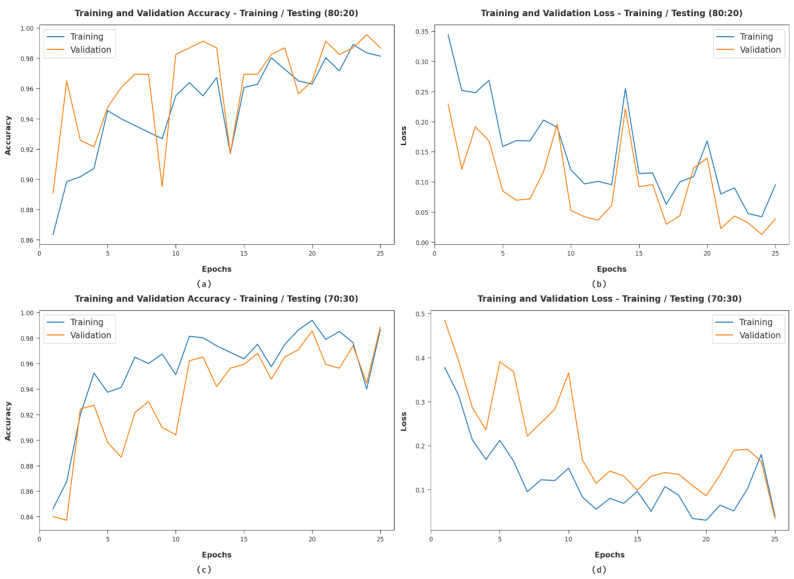
Classification analysis of WDODTL-ODC technique (**a**) 80:20 of accuracy, (**b**) 80:20 of loss, (**c**) 70:30 of accuracy, and (**d**) 70:30 of loss.

Figure 7 offers the accuracy and loss graph analysis of the WDODTL-ODC technique on distinct set of TR/TS datasets. The outcomes outperformed that the accuracy value tends to improve and loss value tends to lesser with enhance in epoch count. It is also observed that the training loss is lower and validation accuracy is high on distinct sets of TR/TS datasets.

**Figure 8:**
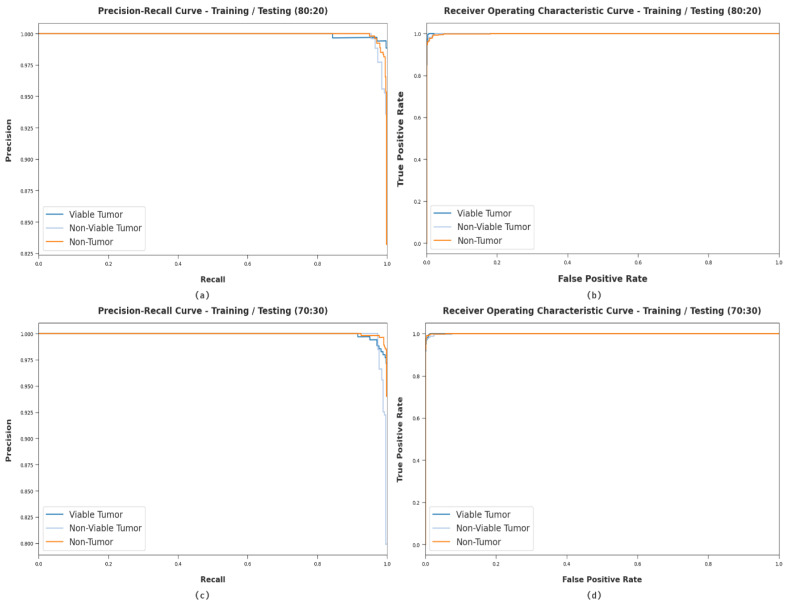
Classification analysis of WDODTL-ODC technique (**a**) 80:20 of precision-recall, (**b**) 80:20 of ROC, (**c**) 70:30 of precision-recall, and (**d**) 70:30 of ROC.

Figure 8 demonstrates the classifier results of the ODCNN-RFIC technique on distinct sets of TR/TS datasets. Figure 8a,c establish the precision recall analysis of the ODCNN-RFIC model under 80:20 and 70:30 datasets. By observing the figure, it can be noticed that the ODCNN-RFIC model has accomplished maximal precision-recall performance under all classes. Lastly, Figure 8b,d illustrate the ROC examination of the ODCNN-RFIC model under 80:20 and 70:30 datasets. The figure indicated that the MFODBN-MDC model has obtained higher ROC under VT, NVT, and NT classes correspondingly.

**Figure 9:**
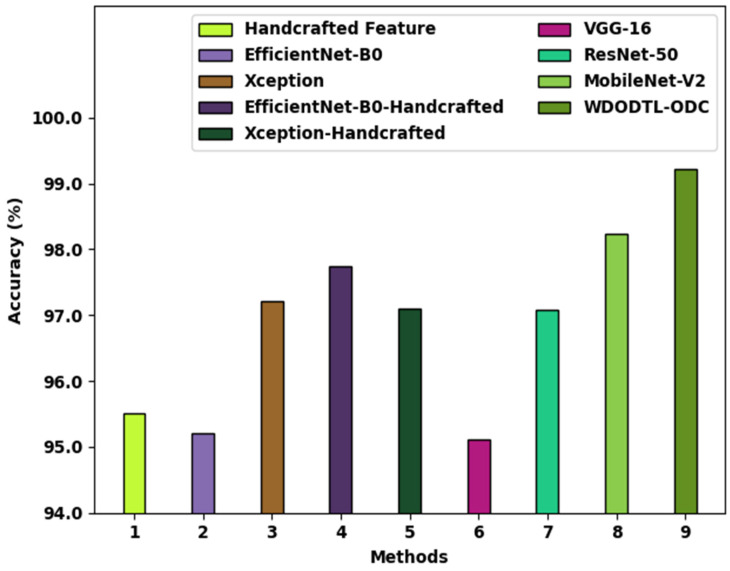
$Accu_{y}$ analysis of WDODTL-ODC technique with existing algorithms.

A detailed comparative analysis of the results offered by the WDODTL-ODC model with recent models is provided in Table 3 [24,25]. Figure 9 reports a brief $accu_{y}$ examination of the WDODTL-ODC model with existing models. The figure implied that the handcrafted feature, EfficientNet-B0, and VGG-16 models have resulted to least classification $accu_{y}$ of 95.50%, 95.20%, and 95.11% respectively. At the same time, the Xception, EfficientNet-B0-Handcrafted, Xception-Handcrafted, and ResNet-50 models have reached slightly enhanced $accu_{y}$ values of 97.22%, 97.74%, 97.10%, and 97.09% respectively. Though the MobileNet-v2 model has shown reasonable performance with $accu_{y}$ of 98.24%, the WDODTL-ODC model has accomplished maximum $accu_{y}$ of 99.22%.

**Figure 10:**
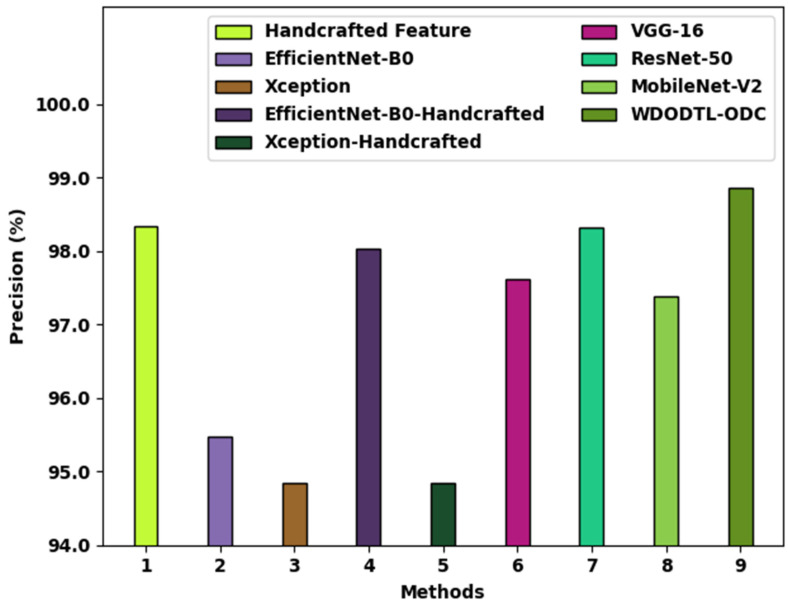
$Prec_{n}$ analysis of WDODTL-ODC approach with existing algorithms.

Figure 10 defines a brief $prec_{n}$ inspection of the WDODTL-ODC approach with existing techniques. The figure implied that the handcrafted feature, EfficientNet-B0, and VGG-16 models have resulted in minimal classification $prec_{n}$ of 98.34%, 95.47%, and 97.61% respectively. At the same time, the Xception, EfficientNet-B0-Handcrafted, Xception-Handcrafted, and ResNet-50 models have reached somewhat improved $prec_{n}$ values of 94.84%, 98.04%, 94.85%, and 98.32% correspondingly. But, the MobileNet-v2 system has shown reasonable performance with $prec_{n}$ of 97.39%, the WDODTL-ODC algorithm has been able higher $prec_{n}$ of 98.86%.

**Figure 11:**
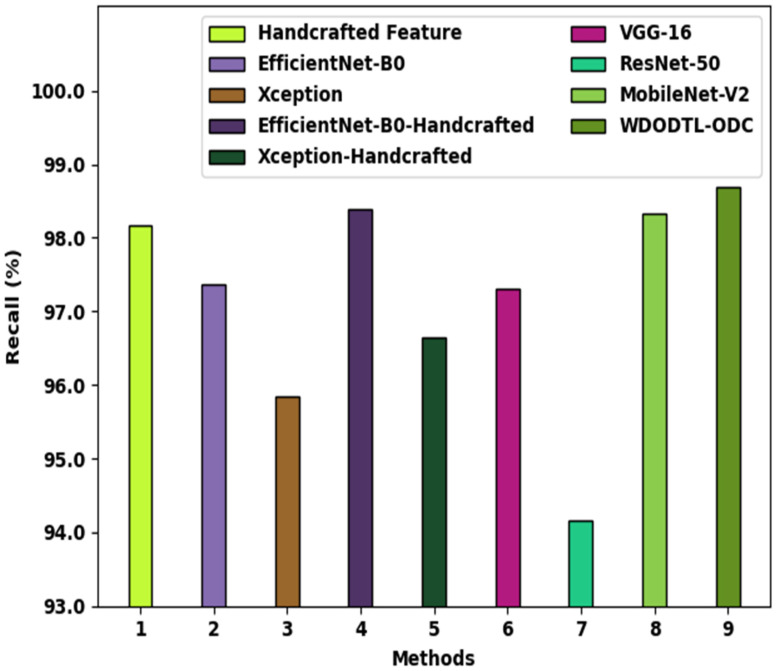
$Reca_{l}$ analysis of WDODTL-ODC approach with existing algorithms.

Figure 11 illustrates a brief $reca_{l}$ investigation of the WDODTL-ODC system with existing techniques. The figure implied that the handcrafted feature, EfficientNet-B0, and VGG-16 models have resulted in least classification $reca_{l}$ of 98.17%, 97.37%, and 97.30% correspondingly. Also, the Xception, EfficientNet-B0-Handcrafted, Xception-Handcrafted, and ResNet-50 models have attained somewhat enhanced $reca_{l}$ values of 95.85%, 98.39%, 96.64%, and 94.16% correspondingly. Eventually, the MobileNet-v2 algorithm has revealed reasonable performance with $reca_{l}$ of 98.33%, the WDODTL-ODC methodology has been able to maximal $reca_{l}$ of 98.69%.

**Figure 12:**
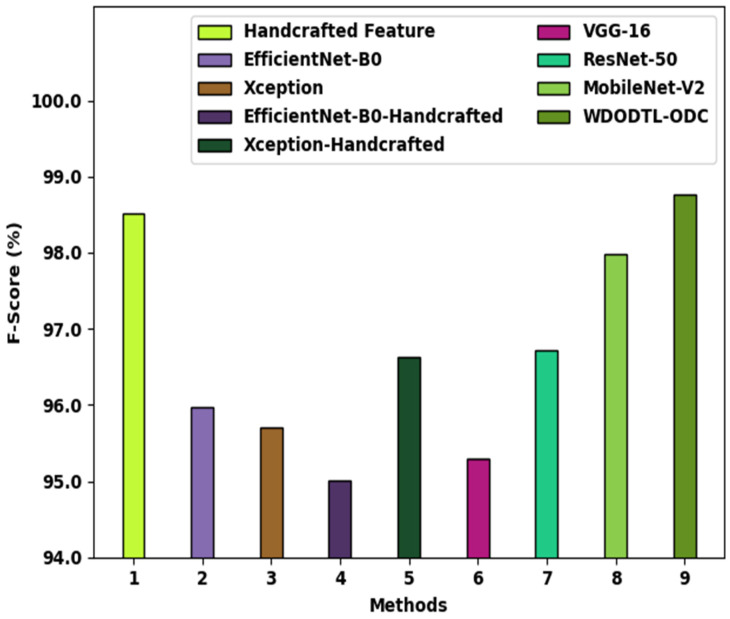
$F_{score}$ analysis of WDODTL-ODC approach with existing algorithms.

Figure 12 showcases a brief $F_{score}$ analysis of the WDODTL-ODC algorithm with existing models. The figure exposed that the handcrafted feature, EfficientNet-B0, and VGG-16 approaches have resulted in least classification $F_{score}$ of 98.51%, 95.97%, and 95.30% correspondingly. Likewise, the Xception, EfficientNet-B0-Handcrafted, Xception-Handcrafted, and ResNet-50 models have reached slightly higher $F_{score}$ values of 95.71%, 95.01%, 96.63%, and 96.71% correspondingly. But the MobileNet-v2 approach has outperformed reasonable performance with $F_{score}$ of 97.98%, the WDODTL-ODC algorithm has accomplished superior $F_{score}$ of 98.77%. The detailed results and discussion confirmed the effectual outcomes of the WDODTL-ODC model over recent models in terms of different evaluation measures. Therefore, the proposed model can be employed for effectual osteosarcoma classification in real time investigation. The enhanced performance of the proposed model is due to the characteristics of SqueezeNet and optimal parameter tuning process using the WDO algorithm.

## 5. Conclusions

In this study, a novel WDODTL-ODC technique has been developed to determine the presence of osteosarcoma in biomedical images. The WDODTL-ODC technique comprises GF based pre-processing and contrast enhancement techniques. In addition, deep transfer learning using SqueezNet model is utilized as feature extractor. At last, the WDO algorithm with DSSAE is employed for classification process. The simulation outcome of the WDODTL-ODC technique is tested using benchmark biomedical images. The simulation outcome demonstrated that the WDODTL-ODC technique outperformed the existing models in the detection of osteosarcoma on biomedical images. Thus, the WDODTL-ODC technique has been exploited for proper identification of osteosarcoma in biomedical images. In future, hybrid DL models can be applied to improve the detection efficiency of the WDODTL-ODC model.

## Figures and Tables

**Table 1:** Result analysis of WDODTL-ODC technique with various measures under 80% of TR and 20% of TS data.

Class Labels	Accuracy	Precision	Recall	F-Score	MCC	Geometric Mean
Training Phase (80%)						
Viable Tumor	99.45	98.49	99.62	99.05	98.67	99.50
Non-Viable Tumor	98.91	97.71	97.71	97.71	96.99	98.49
Non-Tumor	98.36	98.61	97.93	98.27	96.71	98.34
Average	98.91	98.27	98.42	98.34	97.46	98.78
Testing Phase (20%)						
Viable Tumor	100.00	100.00	100.00	100.00	100.00	100.00
Non-Viable Tumor	99.56	97.83	100.00	98.90	98.64	99.73
Non-Tumor	99.56	100.00	99.01	99.50	99.12	99.50
Average	99.71	99.28	99.67	99.47	99.25	99.74

**Table 2:** Result analysis of WDODTL-ODC technique with various measures under 70% of TR and 30% of TS data.

Class Labels	Accuracy	Precision	Recall	F-Score	MCC	Geometric Mean
Training Phase (70%)						
Viable Tumor	99.00	98.30	98.30	98.30	97.59	98.79
Non-Viable Tumor	99.25	99.43	97.19	98.30	97.83	98.51
Non-Tumor	99.00	98.47	99.48	98.97	98.00	99.01
Average	99.08	98.73	98.32	98.52	97.81	98.77
Testing Phase (30%)						
Viable Tumor	99.42	99.09	99.09	99.09	98.66	99.33
Non-Viable Tumor	99.13	98.81	97.65	98.22	97.65	98.63
Non-Tumor	99.13	98.67	99.33	99.00	98.23	99.15
Average	99.22	98.86	98.69	98.77	98.18	99.04

**Table 3:** Comparative analysis of WDODTL-ODC technique with existing approaches.

Methods	Accuracy	Precision	Recall	F-Score
Handcrafted Feature	95.50	98.34	98.17	98.51
EfficientNet-B0	95.20	95.47	97.37	95.97
Xception	97.22	94.84	95.85	95.71
EfficientNet-B0-Handcrafted	97.74	98.04	98.39	95.01
Xception-Handcrafted	97.10	94.85	96.64	96.63
VGG-16	95.11	97.61	97.30	95.30
ResNet-50	97.09	98.32	94.16	96.71
MobileNet-V2	98.24	97.39	98.33	97.98
WDODTL-ODC	99.22	98.86	98.69	98.77

## Data Availability

Data sharing not applicable to this article as no datasets were generated during the current study.
